# Building bridges to integrate care

**DOI:** 10.1186/1472-6963-14-S2-P79

**Published:** 2014-07-07

**Authors:** Tamara Mohammed, Vicky Stergiopoulos, Onil Bhattacharyya, Gary Naglie, Fiona Webster, Elizabeth Mansfield, Jennifer Christian

**Affiliations:** 1 University of Toronto, Toronto, Ontario, Canada

## 

The widespread epidemiological shift from acute to chronic illnesses in the population has been accompanied by an increasing recognition of the need to simultaneously address the interacting physical, mental health and social needs of patients. A fragmented health care system built around single diseases and institutions cannot effectively address these complex needs. Health system reform is necessary to achieve this but is challenged by a lack of evidence on how to effectively restructure the system and care for these complex patients.

Funded by the Ontario Ministry of Health and Long-Term Care (MOHLTC) in Canada, the Building Bridges to Integrate Care (BRIDGES) program at the University of Toronto is jointly led by the Departments of Medicine, Psychiatry and Family and Community Medicine. Led by academics, BRIDGES partners with both the government and providers to address this evidence gap by generating local knowledge on ways in which primary, specialty, hospital and community care may be integrated for patients with complex physical, mental health and social needs. The collaboration with academics supports project teams in developing models that incorporate the best available evidence, engaging in model refinement activities, and rigorously applying qualitative and quantitative evaluation methods. The partnership between academics and government lends credibility to the findings and provides a mechanism through which information from project teams may be consolidated and disseminated to the government to influence work on health system structure and policy reform.

To date, nine models of integrated care delivery have partnered with BRIDGES to form a collaborative that has a common focus, adopts similar evaluation approaches, and shares important lessons. Work with these models has highlighted the difficulties of generating and implementing evidence on care integration. Challenges are present in each stage of model design, implementation, improvement, evaluation, scale and dissemination and are reflected in the form of structural, policy, resource and cultural barriers. In the absence of widespread evidence on how to overcome each of these barriers, the BRIDGES model also provides a knowledge translation platform through which teams interact, share learnings and develop a community of practice where health professionals, researchers and government stakeholders learn from each other’s experiences and work together towards an improved, integrated health care system.

Ongoing work is needed to identify effective models of care for those with complex medical and social needs. BRIDGES is one approach to filling this knowledge gap and generating evidence on effective care integration for this population.

